# Validation of the Brazilian 10-item Cervantes Scale for the assessment of menopausal symptoms

**DOI:** 10.61622/rbgo/2024AO07

**Published:** 2024-03-15

**Authors:** Mona Lúcia Dall’Agno, Charles Francisco Ferreira, Fernanda Vargas Ferreira, Pedro do Valle Teichmann, Jéssica Zandoná, Faustino Ramón Pérez-López, Maria Celeste Osório Wender

**Affiliations:** 1 Universidade Federal do Rio Grande do Sul Porto Alegre RS Brazil Universidade Federal do Rio Grande do Sul, Porto Alegre, RS, Brazil.; 2 Universidad de Zaragoza Zaragoza España Universidad de Zaragoza, Zaragoza, España.

**Keywords:** Climacteric, Quality of life, Surveys and questionnaires, Validation study, Women’s health

## Abstract

**Objective:**

To validate the 10-item Cervantes Scale (CS-10) among Brazilian women.

**Methods:**

This is a cross-sectional observational study involving women in the community aged 40–55 years in the Southern region of Brazil. They completed a general health, habits and socio-demographic questionnaire, the CS-10 and the Women’s Health Questionnaire (WHQ). Women unable to understand the survey, not consenting to participate, or having incapacity imposing difficulties during the completion of the questionnaire were excluded. A Confirmatory Factor Analysis (CFA) was conducted with the AMOS 16.0 software. Chi-square of degrees of freedom (χ2/df), the Comparative Fit Index (CFI), the Tucker-Lewis Index (TLI) and the Root-Mean-Square Error of Approximation (RMSEA) were used as indices of goodness of fit. Cronbach’s alpha coefficient was used for internal consistency.

**Results:**

A total of 422 women were included (premenopausal n=35, perimenopausal n=172, postmenopausal n=215). The CFA for the CS-10 showed a good fit (χ²/df=1.454, CFI=0.989; TLI=0.985; RMSEA=0.033; CI 90%=0.002-0.052; PCLOSE=0.921; Model p=0.049). Good reliability was established in CS-10 and WHQ (Cronbach’s alpha=0.724). Postmenopausal women had higher total CS-10 scores (p≤0.0001), reflecting worse quality of life (QoL) related to menopause symptoms and confirming the greater symptomatology evaluated by high total scores for WHQ found in this population when compared to those in the premenopausal period (p=0.041).

**Conclusion:**

The CS-10 is a consistent tool for health-related QoL in Brazilian mid-aged women.

## Introduction

The world population has experienced the impact of accelerated aging, leading to increased longevity.^(1)^ The consequences of this phenomenon are evident across various segments of society and present growing challenges in the search to maintain and enhance quality of life (QoL).

Globally, rising life expectancy determines approximately half of the women’s lives in the peri- and postmenopausal period, characterized by intense biological, psychological, and social changes. The loss of ovarian function results in hypoestrogenism, responsible for a range of signs and symptoms typical of this period. Alterations in the menstrual cycle and vasomotor symptoms such as hot flushes and night sweats are the most cited; however, other systems and organs suffer from a decrease in serum estrogen that manifests itself as joint pain, sleep and mood disorders, different degrees of sexual dysfunction, genitourinary syndrome of menopause and bone changes.^(2)^ These organic changes in association with the loss of reproductive function and aging itself have a negative impact on QoL.^(3)^

Although the impact of postmenopausal symptoms on women’s QoL seems obvious, there are difficulties in documenting these results due to the subjectivity and multifactorial influences involved. Tools such as questionnaires capable of objectively and quantitatively assessing QoL, allowing comparisons between individuals and populations, play an important role and allow the analysis of QoL to be incorporated into the daily routine of medical consultations and other health actions.^(3)^ There are some specific instruments for climacteric women, which aim to understand, in addition to general aspects, the impact of menopausal symptoms and their relationship with QoL, such as the Cervantes Scale (CS-31), Women’s Health Questionnaire (WHQ), Utian Quality of Life (UQoL), Menopause Quality of Life Questionnaire (MENQoL) and Menopause Rating Scale (MRS).^(3)^ Among the instruments validated for the Brazilian Portuguese language are the CS-31,^(4)^ WHQ,^(5)^ UQoL,^(6)^ and MRS,^(7)^ all composed of an exhaustive and limited number of items from the point of view of applicability. The 10-item Cervantes Scale (CS-10), based on the already established original tool (CS-31),^(8)^ was validated for the Ecuadorian, Colombian, and Portuguese populations.^(9–11)^ It is a questionnaire with 10 items covering three distinct domains, simple and applicable to daily clinical use.^(11)^

This research aimed to validate the CS-10 into Brazilian Portuguese language after applying it to a middle-aged female population, with the aim of providing a concise and reliable tool for measuring the impact of climacteric syndrome on QoL.

## Methods

This is a cross-sectional study, carried out in areas of free access and transit of the population (eg. parks, squares, streets, shopping) in the Southern region of Brazil. The population, involving mid-aged women, is detailed elsewhere.^(12)^

The sample size was estimated by The WinPEPI (PEPI-for-Windows, version 11.65), considering the proportion of climacteric symptoms in middle-aged Colombian women described by Pérez-López et al. (2013)^(11)^ and QoL parameters measured in middle-aged Portuguese women by CS-10 described by Pimenta et al. (2018).^(10)^ Considering the 95% Confidence Interval [95%CI], standard deviation, significance set at 5%, and 10% of losses, 420 participants were necessary.

Sociodemographic characteristics were investigated by a semi-structured questionnaire including age (years), partner status (yes/no), marital status, educational level (total years), parity, professional status, number of people living in the household and family income (in minimum wages). Menopausal status (pre-, peri-, and postmenopausal) was assessed according the Stages of Reproductive Aging Workshop (STRAW+10),^(13)^ and details related to menopausal status, such as menopause age, type of menopause (natural or surgical), pharmacological treatment for menopausal symptoms (yes/no, type) were evaluated. General health and disease data, surgical procedures performed, psychological problems (depression, anxiety), urinary loss, anthropometric data (weight, height and body mass index – BMI, ing kg/m²), as well as life habits (smoke, alcohol and coffee consumption, physical activity) were assessed.

The CS-10^(11)^ is a questionnaire developed based on the original 31-item Cervantes Scale (CS-31).^(8)^ This instrument is used to assess menopausal symptoms and related QoL in mid-aged women. The tool, already validated for the Spanish and the Portuguese (Portugal) languages, considered 10 items from the original scale, and three distinct domains. Each item is scored in a 6-point Likert scale, from 0 ("no symptom") to 5 ("very severe"). The global score is the sum of the scores obtained for each item and can range from 0 to 50. The lower the score, the better the related QoL. There’s no cut off value. This tool has shown adequate psychometric properties in previous studies.^(9,10)^

The Women’s Health Questionnaire (WHQ) was previously developed in England in the 1980s^(14,15)^ and later translated and validated in various languages and populations,^(16)^ including a Brazilian sample.^(5)^ This 36-item instrument is used to assess climacteric symptom experience, associated psychosocial factors, general health, and aging. Also, it can evaluate changes experienced by women during the menopause transition related to QoL.^(16)^ Each item is classified on a four-point scale (Yes, definitely; Yes, sometimes; No, not much; No, not at all). The higher the rating, the greater the suffering and the worse the QoL. There’s no cut off value.

Written informed consent was asked to participants and explained the aims of the study, highlighting that this participation was voluntary and that they could interrupt their collaboration at any time, without consequences. Then participant’s information to the semi-structured questionnaire containing health, habits and sociodemographic data was collected by interview. Finally, women were requested to fill out the CS-10 and the WHQ tools.

Both the original Spanish^(11)^ and the English^(9)^ versions of CS-10 were used for translation and validation. Authorization of the original authors of this instrument was obtained and the first stage of the process - translation and cultural adaptation - was performed using the World Health Organization’s (WHO) instructions.^(17)^ The resulting Brazilian Portuguese translation of this final step was compared with its original version to verify its equivalence.

In order to assess the degree of understanding of the CS-10 by the target population a preliminary study was performed. At the end of this tool, the question "Is there a word that has not been understood?" detects words not understood by women in the early stages of the process. Review of cognitive debriefing results for finalization and proofreading by researchers and experts was also performed. Cultural equivalence was established according to the criteria by Guillemin et al.:^(18)^ at least 85% of the subjects should not show any kind of difficulty to answer each question. The final Brazilian CS-10 questionnaire is shown in chart 1.

**Chart 1:** The final Brazilian 10-item Cervantes Scale (CS-10)

10-item Cervantes Scale (CS-10)					
	No symptoms			Very severe	
1. I have hot flushes (and/or night sweats)	1	2	3	4	5
2. I feel my heart beating quickly and out of control	1	2	3	4	5
3. I cannot get sufficient sleep (difficulty in sleeping)	1	2	3	4	5
4. Aching in muscles and/or joints	1	2	3	4	5
5. I feel tired since i get up (feeling a lack of energy)	1	2	3	4	5
6. I have the perception of being useless	1	2	3	4	5
7. I feel anxious or nervous	1	2	3	4	5
8. I am afraid of performing physical efforts because my urine leaks	1	2	3	4	5
9. I have vaginal discomfort and dryness	1	2	3	4	5
10. I have noticed skin dryness (changes in skin appearance, texture or tone)	1	2	3	4	5

The second stage was the validation of the instrument, corresponding to the statistical analysis of its psychometric properties. The validity occurred via the comparison with a specific reference tool (WHQ), which has been previously validated for the Brazilian Portuguese version.^(5)^

Regarding the data processing, database double entry, review and analysis were performed using the SPSS, version 18.0. [SPSS Inc. Released 2009. PASW Statistics for Windows, Version 18.0. Chicago: SPSS Inc.]. Symmetric data was expressed as mean and standard error of mean (±SEM), or by median and interquartile range ([IQR], 25th–75th percentiles). Categorical variables were described as absolute (n) and relative (n%) frequencies. The Shapiro-Wilk test was used to determine the normality of data distribution. According to this, differences in CS-10 domains and total scores were analyzed with the Kruskal-Wallis test with Dunn *post hoc* test or with the Chi-Square test with adjusted residual analysis for independent samples.

To examine the construct validity of our model for mathematical CS-10, it was calculated the factor loadings of the variables in each model with AMOS 16.0 software. A confirmatory factor analysis (CFA) was conducted. Chi-square of degrees of freedom (χ2/df), the Comparative Fit Index (CFI), the Tucker-Lewis Index (TLI), and the Root-Mean-Square Error of Approximation (RMSEA) were used as indices of the goodness of fit. A χ2/df value below 2 was considered a good model fit; CFI designates the amount of variance and covariance accounted for by the model compared with a baseline model; values higher than 0.90 were considered good. TLI is another incremental fit index and values between 0.90 and 0.95 were considered good. The RMSEA expresses fit per degree of freedom of the model, with values less than 0.10 implying an acceptable model fit and values less than 0.50 indicating a good fit.^(19)^

The psychometric sensitivity was explored through the analysis of both minimum and maximum values and skewness and kurtosis values (acceptable values of skewness fall between − 3 and + 3, and kurtosis is appropriate from a range of − 10 to + 10).^(20)^ The internal consistency (criterion validity) of CS-10 and WHQ instruments was assessed using Cronbach’s alpha coefficients, which was considered good if obtained values were above 0.70.^(21)^ Standardized Root Mean Square Residual (SRMR) was calculated to estimate change in scores standardized relative to variability between the participants, for assessing the internal responsiveness. Because the SRMR is an absolute measure of fit, a value of zero indicates a perfect fit, and a value less than 0.08 is generally considered a good fit.^(22)^ Spearman’s rho (ρ) coefficients were estimated for determining correlations between CS-10 total scores and variables, including scores obtained with the WHQ. The level of significance was set at 5% for all analyses.

Ethical approval was obtained from the Brazil Platform (www.saude.gov.br/plataformabrasil, CAAE reference number 62485816.9.0000.5327) and the Institutional Review Board of the Hospital de Clínicas de Porto Alegre (HCPA, number 16-0621) before data collection begins.

## Results

In total, 422 women met the inclusion criteria of this study, being classified as pre- (n = 35), peri (n = 172), or postmenopausal (n = 215), according to the STRAW+10 criteria. The general characteristics of the surveyed women are presented in table 1.

**Table 1:** Characteristics of all surveyed women (n=422)

Variable		n(%)
Age		
	40 - 44 years	84(19.9)
	45 - 49 years	126(29.9)
	50 - 54 years	173(41.0)
	55 years	39(9.2)
Parity		
	0	55(13.0)
	1	99(23.5)
	≥ 2	268(63.5)
Educational level (years)		
	0 - 6	59(14.0)
	7 - 12	186(44.1)
	≥ 13	177(41.9)
Marital status		
	Married or living with partner	292(69.2)
	Divorced	72(17.1)
	Single	50(11.8)
	Widowed	8(1.9)
	Currently has partner	
	Yes	383(90.5)
	No	40(9.5)
Menopausal status		
	Premenopausal	35(8.3)
	Perimenopausal	172(40.8)
	Postmenopausal	215(50.9)
	Natural menopause	167(39.6)
	Surgical menopause	48(11.4)
Time since menopause onset (years)		5.00 [2.00 - 7.00]
	(minimum - maximum)	(0.00 - 31.00)
Pharmacological treatment for menopause symptoms		
	No	371(87.9)
	Systemic hormone therapy	24(5.7)
	Alternative therapies (herbal teas, phytoestrogens)	24(5.7)
	Psychotropics	1(0.2)
	Topical estrogen	2(0.5)
Habits, lifestyle, health aspects and other issues		
Smoking		
	Current smoking	72(17.1)
	Ex-smoker	100(23.7)
	Non-smoker	250(59.2)
Body mass index		26.05 [23.51 -
	(minimum - maximum)	29.59]
		(16.00 - 54.11)
Physical activity		
	Very active lifestyle	36(8.5)
	Active lifestyle	166(39.3)
	Irregularly active lifestyle	164(38.9)
	Sedentary lifestyle	56(13.3)
Hypertension		55(13.0)
Diabetes		25(5.9)
Psychiatric conditions		114(27.0)
	Depressive disorder	76(18.0)
	Anxiety disorder	30(7.1)
	Bipolar disorder	9(2.1)
	Panic disorder	5(1.2)
	Other disorder	6(1.4)

Data presented as medians (interquartile range [IQR], percentiles 25th and 75th), means ± standard error of means (±SEM) or absolute and relative frequencies [n(n%)]

To achieve the cognitive debriefing, 30 climacteric women (n = 10 in each menopausal status) were included, and no difficulty was observed in the understanding of the proposed CS-10 version (data not shown). The Brazilian Portuguese version of the questionnaire also underwent an assessment by an expert committee (e.g., three gynecologists, one psychologist, and one biologist). There were no suggestions for changes in this version of the CS-10 (Table 2). For the question added at the end of the questionnaire (e.g., "Is there a word that has not been understood?"), the answer "yes" was not marked by any participants in the validation stage (data not shown). The average time for answering the questionnaire was 1 to 2 minutes.

**Table 2:** The 10 items of the short version of the Cervantes Scale (in Brazilian Portuguese and in English)

CS-10 items	Item number according to the CS-31	Brazilian Portuguese version	English version
CS-1	CS-29	Tenho calorões (e/ou suores noturnos)	I have hot flushes (and/or night sweats)
CS-2	CS-23	Sinto o coração acelerado e sem controle	I feel my heart beating quickly and out of control
CS-3	CS-5	Não consigo dormir as horas necessárias (dificuldade no sono)	I cannot get sufficient sleep (difficulty in sleeping)
CS-4	CS-16	Sinto que os músculos e/ou as articulações doem	Aching in muscles and/or joints
CS-5	CS-19	Sinto-me cansada desde que me levanto (falta de energia)	I feel tired since i get up (feeling a lack of energy)
CS-6	CS-21	Tenho a sensação de que não sirvo para nada	I have the perception of being useless
CS-7	CS-2	Sinto-me ansiosa e nervosa	I feel anxious or nervous
CS-8	CS-18	Tenho medo de fazer esforço porque tenho incontinência urinária	I am afraid of performing physical efforts because my urine leaks
CS-9	CS-27	Tenho desconforto e secura vaginal	I have vaginal discomfort and dryness
CS-10	CS-31	Tenho notado a pele mais seca (mudanças na pele, textura ou tom)	I have noticed skin dryness (changes in skin appearance, texture or tone)

CS - Cervantes Scale

**Figure 1:**
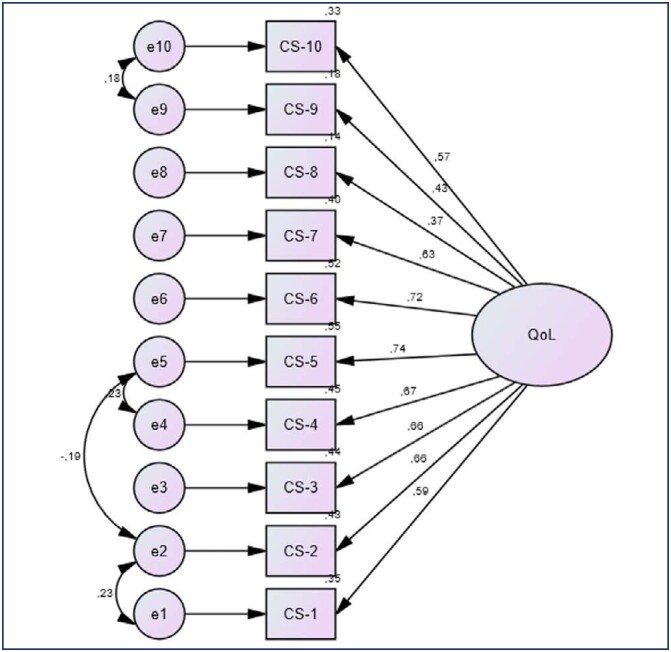
Statistic model- Confirmatory factor loadings of the 10-item Cervantes Scale (AMOS 16.0 software)

The statistical model is shown in figure 1. Each question of the CS-10 was considered a factor loading. The CS-10 measurement model presented a good fit. All factorial weights were above 0.57, except for items 8 (λ=0.37) and 9 (λ=0.43). Likewise, all squared multiple correlations were above 0.14. Kaiser-Meyer-Olkin value (KMO = 0.901) and Bartlet’s test values (Chi-Square = 1344.38, df = 45, p ≤ 0.0001) suggest there is substantial correlation in the data. The Cronbach’s α for the 10 items of the CS-10 showed an excellent reliability (α = 0.857) (data not shown).

Regarding external validity, the model presented a good fit (χ²/df = 1.454, CFI = 0.989; TLI = 0.985; RMSEA = 0.033; CI 90% = 0.002 - 0.052; PCLOSE = 0.921; Model p = 0.049). Moreover, the sensitivity of the items of the CS-10 was adequate; indeed, all items presented values varying between the minimum and maximum, and the skewness and kurtosis showed adequate values (data not shown). Briefly, there were no missing values, no ceiling and low floor effects was observed (item CS-8, |kurtosis| = 8.40; |skewness| = 2.99). SRMR (= 0.0292) also presented a good fit.

The CS-10 comparisons among menopausal status were conducted with continuous and categorical analysis. Medians [IQR] for hot flushes (and/or night sweats) frequencies were different among all groups, since the premenopausal group displayed the lowest values (0.00 [0.00 - 1.00]), the perimenopausal group displayed intermediate values (1.00 [0.00 - 3.00]), and the postmenopausal group displayed the highest values (2.00 [0.00 - 4.00]) (Kruskal-Wallis with Dunn *post hoc*, p ≤ 0.0001). Medians [IQR] for heart beating quickly frequencies were different among among all groups, since the premenopausal group displayed lower values (0.00 [0.00 - 1.00]) in relation to both, the peri- (1.00 [0.00 - 3.00]) and the postmenopausal (1.00 [0.00 - 3.00]) groups (Kruskal-Wallis with Dunn *post hoc*, p = 0.014). Medians [IQR] for vaginal discomfort/dryness and skin dryness was lower in the pre- (0.00 [0.00 - 0.00] and 1.00 [0.00 - 2.00], respectively) and the perimenopausal groups (0.00 [0.00 - 1.00] and 1.00 [0.00 - 3.00], respectively), in relation to the postmenopausal group (1.00 [0.00 - 3.00] and 3.00 [0.00 - 4.00], respectively) (Kruskal-Wallis with Dunn *post hoc*, p ≤ 0.0001, for both). When comparing the CS-10 total scores among groups, the median [IQR] of peri- (13.00 [6.00-24.00]) and postmenopausal (15.00 [8.50-48.00]) group was higher in relation to the pre-menopausal (9.00 [4.00-15.00]) (Kruskal-Wallis with Dunn *post hoc*, p ≤ 0.0001). When comparing the WHQ total score among groups, the median [IQR] of postmenopausal (42.00 [28.50-52.50]) group was higher in relation to the pre-menopausal (29.00 [19.00-41.50]) group (Kruskal-Wallis with Dunn *post hoc*, p = 0.041). WHQ total score from the perimenopausal group did not differ neither from the pre- nor from the postmenopausal groups (Kruskal-Wallis with Dunn post hoc, p > 0.05 for both). For both scales, a higher total score is equivalent to a worse quality of life (Table 3).

**Table 3:** The 10 items of the short version of the Cervantes Scale

Variable	Total n=422	Premenopausal n=35	Perimenopausal n=172	Postmenopausal n=215	*p-value
(CS-1) I have hot flushes (and/or night sweats)	1.00 [0.00 - 3.00]	0.00 [0.00 - 1.00]a	1.00 [0.00 - 3.00]b	2.00 [0.00 - 4.00]c	≤ 0.0001
(minimum- maximum)	(0.00 - 5.00)	(0.00 - 4.00)	(0.00 - 5.00)	(0.00 - 5.00)	
(CS-2) I feel my heart beating quickly and out of control	1.00 [0.00 - 3.00]	0.00 [0.00 - 1.00]a	1.00 [0.00 - 3.00]b	1.00 [0.00 - 3.00]b	0.014
(minimum- maximum)	(0.00 - 5.00)	(0.00 - 3.00)	(0.00 - 5.00)	(0.00 - 5.00)	
(CS-3) I cannot get sufficient sleep (difficulty in sleeping)	1.00 [0.00 - 4.00]	0.00 [0.00 - 3.00]	1.00 [0.00 - 3.00]	2.00 [0.00 - 4.00]	0.159
(minimum- maximum)	(0.00 - 5.00)	(0.00 - 5.00)	(0.00 - 5.00)	(0.00 - 5.00)	
(CS-4) Aching in muscles and/or joints	2.00 [0.00 - 4.00]	1.00 [0.00 - 3.00]	2.00 [0.00 - 4.00]	3.00 [1.00 - 4.00]	0.113
(minimum- maximum)	(0.00 - 5.00)	(0.00 - 5.00)	(0.00 - 5.00)	(0.00 - 5.00)	
(CS-5) I feel tired since i get up (feeling a lack of energy)	1.00 [0.00 - 3.00]	1.00 [0.00 - 2.00]	1.00 [0.00 - 3.00]	1.00 [0.00 - 3.00]	0.255
(minimum- maximum)	(0.00 - 5.00)	(0.00 - 4.00)	(0.00 - 5.00)	(0.00 - 5.00)	
(CS-6) I have the perception of being useless	0.00 [0.00 - 2.00]	0.00 [0.00 - 0.50]	0.00 [0.00 - 2.00]	0.00 [0.00 - 2.50]	0.130
(minimum- maximum)	(0.00 - 5.00)	(0.00 - 4.00)	(0.00 - 5.00)	(0.00 - 5.00)	
(CS-7) I feel anxious or nervous	3.00 [1.00 - 4.00]	2.00 [1.00 - 3.00]	3.00 [1.00 - 4.00]	2.00 [1.00 - 4.00]	0.130
(minimum- maximum)	(0.00 - 5.00)	(0.00 - 5.00)	(0.00 - 5.00)	(0.00 - 5.00)	
(CS-8) I am afraid of performing physical efforts because my urine leaks	0.00 [0.00 - 0.00]	0.00 [0.00 - 0.00]	0.00 [0.00 - 0.00]	0.00 [0.00 - 0.00]	0.193
(minimum- maximum)	(0.00 - 5.00)	(0.00 - 3.00)	(0.00 - 5.00)	(0.00 - 5.00)	
(CS-9) I have vaginal discomfort and dryness	0.00 [0.00 - 2.00]	0.00 [0.00 - 0.00]a	0.00 [0.00 - 1.00]a	1.00 [0.00 - 3.00]b	≤ 0.0001
(minimum- maximum)	(0.00 - 5.00)	(0.00 - 5.00)	(0.00 - 5.00)	(0.00 - 5.00)	
(CS-10) I have noticed skin dryness (skin appearance, texture or tone)	2.00 [0.00 - 4.00]	1.00 [0.00 - 2.00]a	1.00 [0.00 - 3.00]a	3.00 [0.00 - 4.00]b	≤ 0.0001
	(0.00 - 5.00)	(0.00 - 5.00)	(0.00 - 5.00)	(0.00 - 5.00)	
Total CS-10 score	14.00 [6.00-24.00]	9.00 [4.00-15.00]a	13.00 [6.00-24.00]b	15.00 [8.50-27.00]b	≤ 0.0001
(minimum- maximum)	(0.00 - 48.00)	(0.00 - 28.00)	(0.00 - 46.00)	(0.00 - 48.00)	
Total Women’s Health Questionnaire score	40.00 [26.00-52.00]	29.00[19.00-41.50]a	39.00[26.00-53.00]ab	42.00[28.50-52.50]b	0.041
(minimum- maximum)	(0.00 - 102.00)	(3.00 - 63.00)	(0.00 - 92.00)	(0.00 - 102.00)	

Data presented as medians (interquartile range [IQR], percentiles 25th and 75th), or absolute and relative frequencies [n(n%)]. Legend: p - significance index. CS - Cervantes scale.

* Kruskal-Wallis test with Dunn post hoc, or Chi-Square test with adjusted residual analysis. Significance set at 5% for all analysis. Bold numbers indicate association between categories/variables. ab Different letters indicate significant differences among menopausal group

Severe hot flushes were associated with the postmenopausal group (19.5%), while no hot flush symptom had a higher frequency in pre- (71.4%) and perimenopausal (49.4%) groups (Chi-Square test, p=0.001). In the same way, the postmenopausal group was associated with skin dryness (appearance, texture, or tone) (20.0%), while pre- (48.6%) and perimenopausal (40.1%) groups were not (Chi-Square test, p=0.003). Both pre- and post-menopausal groups were associated with feeling anxious or nervous (28.6% and 18.6%, respectively) (Chi-Square test, p=0.003). In addition, both pre- and perimenopausal groups were associated with no vaginal discomfort and dryness (82.9% and 66.9%, respectively) (Table 4).

**Table 4:** The 10 items of the short version of the Cervantes Scale

Variable		Total n=422 n(%)	Premenopausal n=35 n(%)	Perimenopausal n=172 n(%)	Postmenopausal n=215 n(%)	*p-value
(CS-1) I have hot flushes (and/or night sweats)						0.001
	0 (no symptom)	184(43.6)	25(71.4)	85(49.4)	74(34.4)	
	1	42(10.0)	5(14.3)	18(10.5)	19(8.8)	
	2	43(10.2)	2(5.7)	16(9.3)	25(11.6)	
	3	64(15.2)	2(5.7)	26(15.1)	36(16.7)	
	4	31(7.3)	1(2.9)	11(6.4)	19(8.8)	
	5 (very severe)	58(13.7)	0(0.0)	16(9.3)	42(19.5)	
(CS-2) I feel my heart beating quickly and out of control						0.077
	0 (no symptom)	205(48.6)	25(71.4)	79(45.9)	101(47.0)	
	1	62(14.7)	3(8.6)	27(15.7)	32(14.9)	
	2	47(11.1)	3(8.6)	21(12.2)	23(10.7)	
	3	53(12.6)	4(11.4)	27(15.7)	22(10.2)	
	4	23(5.5)	0(0.0)	9(5.2)	14(6.5)	
	5 (very severe)	32(7.6)	0(0.0)	9(5.2)	23(10.7)	
(CS-3) I cannot get sufficient sleep (difficulty in sleeping)						0.473
	0 (no symptom)	171(40.5)	18(51.4)	75(43.6)	78(36.3)	
	1	45(10.7)	2(5.7)	16(9.3)	27(12.6)	
	2	49(11.6)	5(14.3)	17(9.9)	27(12.6)	
	3	51(12.1)	5(14.3)	22(12.8)	24(11.2)	
	4	35(8.3)	2(5.7)	17(9.9)	16(7.4)	
	5 (very severe)	71(16.8)	3(8.6)	25(14.5)	43(20.0)	
(CS-4) Aching in muscles and/or joints						0.308
	0 (no symptom)	116(27.5)	12(34.3)	53(30.8)	51(23.7)	
	1	61(14.5)	6(17.1)	26(15.1)	29(13.5)	
	2	41(9.7)	2(5.7)	14(8.1)	25(11.6)	
	3	64(15.2)	7(20.0)	25(14.5)	32(14.9)	
	4	51(12.1)	6(17.1)	16(9.3)	29(13.5)	
	5 (very severe)	89(21.1)	2(5.7)	38(22.1)	49(22.8)	
(CS-5) I feel tired since i get up (feeling a lack of energy)						0.565
	0 (no symptom)	181(42.9)	17(48.6)	76(44.2)	88(40.9)	
	1	45(10.7)	5(14.3)	16(9.3)	24(11.2)	
	2	48(11.4)	6(17.1)	21(12.2)	21(9.8)	
	3	55(13.0)	3(8.6)	21(12.2)	31(14.4)	
	4	44 (10.4)	4(11.4)	16(9.3)	24(11.2)	
	5 (very severe)	49(11.6)	0(0.0)	22(12.8)	27(12.6)	
(CS-6) I have the perception of being useless						0.398
	0 (no symptom)	256(60.7)	26(74.3)	102(59.3)	128(59.5)	
	1	38(9.0)	4(11.4)	18(10.5)	16(7.4)	
	2	33(7.8)	2(5.7)	14(8.1)	17(7.9)	
	3	43(10.2)	1(2.9)	14(8.1)	28(13.0)	
	4	22(5.2)	2(5.7)	11(6.4)	9(4.2)	
	5 (very severe)	30(7.1)	0(0.0)	13(7.6)	17(7.9)	
(CS-7) I feel anxious or nervous						0.003
	0 (no symptom)	82(19.4)	6(17.1)	35(20.9)	40(18.6)	
	1	64(15.2)	8(22.9)	28(16.3)	28(13.0)	
	2	59(14.0)	10(28.6)	9(5.2)	40(18.6)	
	3	76(18.0)	6(17.1)	33(19.2)	37 (17.2)	
	4	53(12.6)	3(8.6)	25(14.5)	25(11.6)	
	5 (very severe)	88(20.9)	2(5.7)	41(23.8)	45(20.9)	
(CS-8) I am afraid of performing physical efforts because my urine						0.305
leaks		353(83.6)	33(94.3)	144(83.7)	176(81.9)	
	0 (no symptom)	27(6.4)	1(2.9)	8(4.7)	18(8.4)	
	1	12(2.8)	0(0.0)	3(1.7)	9(4.2)	
	2	14(3.3)	1(2.9)	9(5.2)	4(1.9)	
	3	7(1.7)	0(0.0)	3(1.7)	4(1.9)	
	4	9(2.1)	0(0.0)	5(2.9)	4(1.9)	
	5 (very severe)					
(CS-9) I have vaginal discomfort and dryness						0.002
	0 (no symptom)	249(59.0)	29(82.9)	115(66.9)	105(48.8)	
	1	46(10.9)	0(0.0)	18(10.5)	28(13.0)	
	2	37(8.8)	4(11.4)	9(5.2)	24(11.2)	
	3	40(9.5)	0(0.0)	15(8.7)	25(11.6)	
	4	15(3.6)	1(2.9)	3(1.7)	11(5.1)	
	5 (very severe)	35(8.3)	1(2.9)	12(7.0)	22(10.2)	
(CS-10) I have noticed skin dryness (skin appearance, texture or tone)						0.003
	0 (no symptom)	144(34.1)	17(48.6)	69(40.1)	58(27.0)	
	1	46(10.9)	5(14.3)	22(12.8)	19(8.8)	
	2	52(12.6)	6(17.1)	18(10.5)	29(13.5)	
	3	68(16.1)	2(5.7)	25(14.5)	41(19.1)	
	4	52(12.3)	3(8.6)	24(14.0)	25(11.6)	
	5 (very severe)	59(14.0)	2(5.7)	14(8.1)	43(20.0)	

Data presented as absolute and relative frequencies [n(n%)]; p - significance index. CS - Cervantes scale;

* Chi-Square test with adjusted residual analysis; Significance set at 5% for all analysis; Bold numbers indicate association between categories/variables

The Spearman’s correlation between the CS-10 and the WHQ total scores (to assert criterion validity) presented a positive and significant, and strong association (ρ = 0.639, p ≤ 0.0001). In addition, both questionnaires’ total scores displayed substantial reliability (α = 0.724).

## Discussion

The CS-10 was translated, culturally adapted and validated into Brazilian Portuguese language and we demonstrated that the instrument presents good internal consistency, validity and reliability for assessing QoL.

In the CFA and assessment of the internal consistency, we found the validity of the CS-10 to be satisfactory in a group of 422 women according to previous validation studies.^(9–11)^ The CS-10 CFA showed an acceptable fit (χ^2^/df=1.454, CFI=0.989, TLI=0.985, RMSEA=0.033, CI 90%=0.002–0.052, p≤0.001) and appropriate values were evidenced in terms of both factorial weights, as well as regarding the multiple squared correlations. Also, this study indicates that the Brazilian version of CS-10 has excellent reliability (Cronbach’s alpha=0.857) as a health-related QoL instrument in mid-aged women, and climacteric symptoms and domains measured by WHQ reinforced these CS-10 properties.

In this study, the three most frequent menopause symptoms were "feelings of anxiety and nervousness" (reported by 80.6% of women in this sample), "joint and muscle aches" (72.5%), and "skin dryness" (65.9%). Moreover, the three most severe menopause symptoms (items rated 4 or above) were "joint and muscle aches" (33.2%), "skin dryness" (26.3%), and "sleep-related problems" (25.1%). Different results were obtained in other studies including Colombian, Ecuadorian, and Portuguese women who were assessed with the CS-10.^(9–11)^ In those countries, the most prevalent menopausal symptom was "muscle and joint pains". Despite that, the other symptoms included in the most frequent symptoms reported by the Brazilian sample displayed similar results to previous studies. The second most prevalent symptom in Portuguese women was "feelings of anxiety and nervousness", and the third in Ecuadorian women were also "skin dryness". Furthermore, when comparing the most severe symptoms, we understand the results to be comparable, as the three samples obtained at least an equal report of the three symptoms mentioned as the most severe.

The differences in symptom expression when comparing the present study and the Colombian, Ecuadorian, and Portuguese studies^(9–11)^ may be associated with the menopause-related characteristics of the sample. The majority of Brazilian women were postmenopausal, as were the Ecuadorian and Colombian. However, the Portuguese sample was mostly perimenopausal. Previous research has shown that the prevalence of menopause-related symptoms (ie, hot flushes)^(21,23)^ is higher in post menopause than in perimenopause women, corroborating the finding that almost only postmenopausal women related "hot flushes" in the Brazilian sample, even though it is not the most common symptom in this population. The low prevalence of hot flushes can be explained by the time since menopause of the sample. The median [IQR] time since menopause onset was 5 years, and at this time other later symptoms can predominate (e.g. vaginal and skin dryness). Furthermore, 12.1% of the Brazilian women were being treated for vasomotor symptoms at the time of the interview.

Menopausal status defines an impact on QoL due to biological, social, cultural, physical, and psychological aspects.^(24)^ As expected, total CS-10 scores were positively associated with menopausal status and aging. The median [IQR] of the postmenopausal (15.00 [8.50-48.00]) was higher than the peri- (13.00 [6.00-24.00]) and the premenopausal (9.00 [4.00-15.00]) groups. No difference was observed in CS-10 total score between the peri- and the postmenopausal groups. The WHQ is a health related QoL tool and can also assess menopause symptoms. The higher the WHQ total score, the worse the QoL. The same occurs with the CS-10 total score, reflecting the positive and significant strong association between the two questionnaires. Considering these results, the Brazilian version of the CS-10 is a consistent tool for the assessment of menopause symptoms and QoL related in mid-aged Brazilian women. This new tool can be used in different ethnic populations and medical conditions with good reliability values.^(9–11)^ It was developed as a shorter and concise alternative, with the same psychometric properties as the original 31 items.^(4,8)^

Certain limitations should be considered. Due to the methodology of this research, it was not possible to carry out a test-retest as part of the validation process. This is a cross-sectional study and the interviews were conducted with women from the community and in public places, which made it impossible to conduct a new interview with the same participants.

Should be mentioned that the present study used a sample in which 27% self-reported psychological conditions. Information about treatment is lacking and could impact the symptom’s CS-10 scores. Furthermore, the general information collected through self-reporting questionnaires can lack diagnostic precision.^(25)^ This may occur due to the difficulty of the subjects understanding the questions, although, as mentioned earlier, it was not the case in the present study (no difficulty was observed in understanding the proposed CS-10 version). Also, there is difficulty in measuring the extent to which symptoms lead to stress or suffering for each of the subjects. We believe that self-reporting instruments lead to more reliable answers since the intimacy of the participant is preserved when responding.

Another important point is that the sexuality domain was not included in CS-10, despite being an important aspect of QoL and a relevant item to be assessed during menopause. However, the female sexual function can be evaluated with a more specific, simple, standardized, and comprehensive tool such as the popular Female Sexual Function Index.^(12,26)^

This short version scale should be intended for menopausal clinical symptom assessment. Sexuality should be assessed with specific tools, like FSFI-6 which can provide quick and accurate information and is already validated to the Brazilian mid-age women population.^(12)^ Finally, there is no cut-off to categorize the total score of CS-10 into degrees of QoL, in spite of the fact that there isn’t such a categorization. We believe the applicability of this tool can be a priority in clinical practice, to compare the same women in different moments and rate the health related QoL (e.g. evaluate the response to treatment).

## Conclusion

The translated and adapted to Brazilian culture CS-10 is a consistent tool for menopause symptoms and QoL assessment in mid-aged women. Although there is no background data related to the QoL of Brazilian mid-aged women assessed with the CS-10, our current results seem to be comparable to reported results from other countries. The use of the same validated instruments allows a transcultural comparison, contributing to valuable knowledge on how women from different countries experience menopausal symptoms. The CS-10, already used in middle-aged women of Colombia, Ecuador, and Portugal, demonstrated good psychometric properties when applied to middle-aged Brazilian women and, therefore, can be used in clinical and community settings.
